# SLE and Serum Complement: Causative, Concomitant or Coincidental?

**DOI:** 10.2174/1874312901812010171

**Published:** 2018-09-18

**Authors:** Vaneet Sandhu, Michele Quan

**Affiliations:** 1 Division of Rheumatology, Loma Linda University Medical Center, Loma Linda, CA, USA; 2 Department of Internal Medicine, Arrowhead Regional Medical Center, Colton, CA, USA

SLE and Serum Complement: Causative, Concomitant or Coincidental?

The Open Rheumatology Journal, **2017**, 11: 113-122

**The revised last paragraph of conclusion in abstract is mentioned below:**

It is clinically important to find novel ways to assess disease activity in SLE. Increased serum levels of cell-bound complement activation products may more accurately reflect disease activity than conventional serum C3 and C4 monitoring.

**The original last paragraph of conclusion provided was:**

It is clinically important to find novel ways to assess disease activity in SLE. Reduced serum levels of cell-bound complement activation products may more accurately reflect disease activity than conventional serum C3 and C4 monitoring.

**The revised last paragraph of conclusion is mentioned below:**

With recent studies demonstrating that increased levels of serum cell-bound complement activation products may more accurately reflect disease activity than conventional complement C3 and C4 monitoring, we believe that this is an important area for future SLE research and look forward to further studies on research in the complement in SLE.

**The original last paragraph of conclusion provided was:**

With recent studies demonstrating that reduced levels of serum cell-bound complement activation products may more accurately reflect disease activity than conventional complement C3 and C4 monitoring, we believe that this is an important area for future SLE research and look forward to further studies on research in the complement in SLE.

